# Probing chromatin accessibility with small molecule DNA intercalation and nanopore sequencing

**DOI:** 10.1101/2024.03.20.585815

**Published:** 2024-03-22

**Authors:** Gali Bai, Namrita Dhillon, Colette Felton, Brett Meissner, Brandon Saint-John, Robert Shelansky, Elliot Meyerson, Eva Hrabeta-Robinson, Babak Hodjat, Hinrich Boeger, Angela N. Brooks

**Affiliations:** 1 Department of Biomolecular Engineering, University of California, Santa Cruz, Santa Cruz, California, 95064, United States of America; 2 Department of Molecular, Cell, and Developmental Biology, University of California, Santa Cruz, Santa Cruz, California, 95064, United States of America; 3 Cognizant AI Labs, San Francisco, California, 94105, United States of America

## Abstract

Genome-wide identification of chromatin organization and structure has been generally probed by measuring accessibility of the underlying DNA to nucleases or methyltransferases. These methods either only observe the positioning of a single nucleosome or rely on large enzymes to modify or cleave the DNA. We developed adduct sequencing (Add-seq), a method to probe chromatin accessibility by treating chromatin with the small molecule angelicin, which preferentially intercalates into DNA not bound to core nucleosomes. We show that Nanopore sequencing of the angelicin-modified DNA is possible and allows visualization and analysis of long single molecules with distinct chromatin structure. The angelicin modification can be detected from the Nanopore current signal data using a neural network model trained on unmodified and modified chromatin-free DNA. Applying Add-seq to *Saccharomyces cerevisiae* nuclei, we identified expected patterns of accessibility around annotated gene loci in yeast. We also identify individual clusters of single molecule reads displaying different chromatin structure at specific yeast loci, which demonstrates heterogeneity in the chromatin structure of the yeast population. Thus, using Add-seq, we are able to profile DNA accessibility in the yeast genome across long molecules.

## GRAPHICAL ABSTRACT

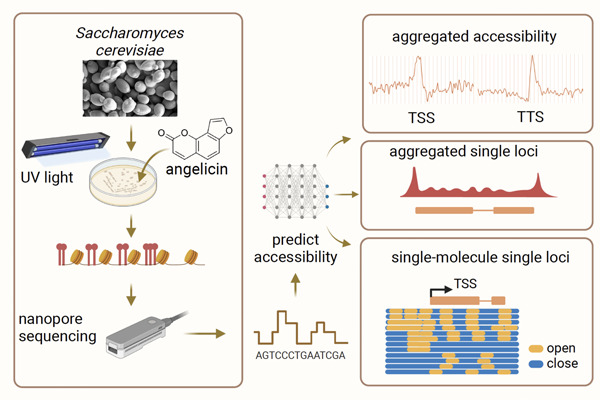

## INTRODUCTION

DNA in all eukaryotic cells is packaged into nucleosomes. This nucleoprotein complex together with DNA binding proteins and RNA comprises chromatin. The dynamic and variable nature of chromatin regulates all DNA-centric processes and plays a vital role in cell growth, differentiation, and development. Nucleosomes are composed of approximately 147bp (~ 1.7 turns) of DNA wrapped around a central histone protein octamer. Arrays of nucleosomes separated by ~20–90 bp of linker DNA appear as beads on a string in electron micrographs (1). Nucleosomes block access of DNA binding factors to the underlying DNA and impede transcription, replication, DNA repair and recombination machineries (2). The distribution of nucleosomes across the genome is not uniform and varies significantly between open and closed chromatin. There is also considerable heterogeneity in nucleosome distribution at different gene loci in open chromatin and also within each gene (3). This chromatin structure varies with growth conditions, differentiation, and development (4). Thus, knowledge of the dynamic chromatin landscape can yield important insights into development, disease, and drug response.

Assays to determine nucleosome distribution at specific gene loci were developed soon after the discovery of the nucleosome (5). The original assays probed for accessibility of chromatin to DNA endonucleases that mostly cleave linker DNA (6, 7). These were subsequently adapted to genome-wide nucleosome distribution studies using short-read Illumina sequencing leading to MNase-seq (8), DNAse-seq (9), and ATAC-seq (10) among others. While nucleosome distribution profiles from short-read data have been vital to our understanding of chromatin structure and function, they only provide an aggregate view of nucleosome distribution across all cells in the population. A granular view of the heterogeneity in nucleosome spacing in individual cells is lacking in these data. Also absent is a view of coordination of nucleosome organization across long genomic distances. short-read data also suffer from biases introduced by PCR amplification, read mapping, and DNA fragmentation (11).

A more recent advancement in sequencing was the development of long-read nanopore sequencing technology, where an electrical current is passed across a biological pore embedded in a lipid bilayer. As single-stranded DNA is channeled through the pore by a motor protein, the electrical current undergoes shifts based on the sequence of the six bases of DNA (k-mer) present in the pore at any given time (12). Recent advances in nanopore sequencing technology where electrical shifts generated by modified DNA bases can also be detected have led to the development of single molecule long-read assays to map chromatin accessibility using DNA methyltransferases (MTase) (13). These long-read sequencing approaches allow for the detection of modified DNA without the bias of PCR amplification and can also detect endogenous DNA modifications such as 6mA and 5mC (14–17). Data from these methods have yielded novel insights into single-cell nucleosome distribution in the genome and gene regulation.

Despite the promise of MTase assays to map nucleosome occupancy, they all rely on the ability of the MTase, a bulky protein with an average atomic mass of 38kD, to access linker DNA (the short spans of DNA between individual nucleosomes). To overcome the issue of labeling short linker regions, we have developed a method to map accessible chromatin using the small molecule, angelicin, which has an atomic mass of 186. Angelicin is a furocoumarin, a class of molecule that covalently binds DNA pyrimidine bases when exposed to UV-A light. The most widely used furocoumarin in structural DNA analysis is the DNA crosslinking agent psoralen (18). Unlike the crosslinked di-adducts generated by psoralen, angelicin is thought to form only monoadducts within a single strand of DNA, i.e. it is linked to one DNA strand alone (19, 20). Angelicin was also shown to intercalate with a sequence preference of 5’-TA > 5’AT >> 5’TG > 5’GT bases (21). The use of a monoadduct to modify DNA allows us to generate single stranded DNA required for nanopore sequencing and avoids damaging the high molecular weight DNA needed for long-read sequencing. Here we report a method called Add-seq, for adduct sequencing, that utilizes angelicin to map chromatin accessilbilty using nanopore sequencing. We show that intercalation of angelicin causes a detectable shift in the nanopore current signal and have developed a neural network approach to predict chromatin structure from this signal data. We show that angelicin modification data recapitulates known patterns of chromatin structure and identifies heterogenous single-molecule chromatin profiles and regulatory patterns at a single locus.

## MATERIAL AND METHODS

### Yeast strains and culture

The *Saccharomyces cerevisiae* strain ys18 (MA Talpha his3–11 his3–15 leu2–3 leu2–112 can1–100 ura3∆5) (S288C derivative) was used in this study. Cells were grown in YPD (1% yeast extract, 2% peptone, 2% dextrose) at 30°C.

### Yeast nuclei isolation

Yeast nuclei isolation was carried out as described previously (22).

### Angelicin modification of yeast and genomic DNA extraction

Yeast chromatin was modified with angelicin using purified nuclei. One aliquot of nuclei (from ~5 X 10^8 cells) was resuspended in 0.4 mL of angelicin modification buffer (10mM Tris-HCl, 10mM NaCl, 0.1mM EDTA, pH 7.4). The nuclei suspension was divided into 2 wells of a 6-well cell culture plate and placed on ice. 10 uL of a 2mg/ml angelicin stock (500uM) (SIGMA-A0956) were added to each of the wells. Angelicin is photolytic, and care should be taken to ensure samples incubating with angelicin are kept away from direct UV-light before and after crosslinking. The plate was swirled a few times to mix the angelicin and incubated in the dark on ice for 5 minutes. While ensuring the culture plate remained nested in ice, the plate was exposed to 365nm UVA light (Stratagene UV Stratalinker 2400, power 5.0) for 5 minutes followed by a 5-minute incubation in ice. This UV exposure process was repeated for a total of 7 times. Contents of both wells were pooled into a fresh low-adhesion tube (EPPENDORF-022431021) and both wells were washed with 100 uL of ice-cold angelicin modification buffer and added to the same low-adhesion tube to maximize nuclei retrieval. High molecular weight DNA was purified using the NEB Monarch HMW DNA Extraction kit for tissue (T3060L). The use of wide-bore pipette tips when working directly with long DNA massively improves the length of the purified library. Positive and negative control data for the neural network training were generated from purified high molecular weight DNA that was incubated with or without 500uM angelicin respectively followed by UV treatment as described above.

### Oxford nanopore sequencing

3–4 micrograms of DNA were used to prepare genomic libraries for sequencing with Oxford Nanopore Technologies (ONT) SQK-LSK110 kits for use with R9.4.1 (FLO-MIN106) flowcells. ~1.5 micrograms of the library were loaded onto flowcells, and all library sequencing was undertaken on a MinION for 24 hours each with MUX scanning every 6 hours to extend the life of the flow cell.

### Basecalling and pre-processing sequencing data

The data was basecalled with guppy v4.4.0 and the reads aligned to the sacCer3 genome with minimap2 v2.26 (23, 24). Secondary and supplementary reads were then filtered and aligned reads were sorted and indexed with samtools v1.13. Nanopolish eventalign v0.14.0 was run to align signals to the kmers (25).

### Quantification of angelicin modification

To assay the extent of angelicin modification, we selected the restriction endonuclease BciVI as its recognition sequence harbors a central ‘AT’ motif that becomes unrecognizable in the presence of an angelicin modification. Plasmid vector pBluescript SK+ (https://www.addgene.org/vector-database/1951/) was modified with 0, 100, and 500 uM angelicin prior to digestion with BciVI (NEB). 2.5 micrograms of DNA modified at each angelicin concentration were cut with either 4 or 8 units of restriction enzyme. Digests were analyzed on a 4150 TapeStation (Agilent) using a D5000 Screentape.

### Alkaline agarose gel electrophoresis

Agarose gel electrophoresis was performed on pBlueScript DNA modified with 0, 100, and 500 uM angelicin and digested with Not1 according to (32). DNA was visualized and documented on a BioRad ChemiDoc XRS imager after ethidium bromide staining and destaining.

### Identification of kmer signal distribution peaks and informative kmers

We took the mean signal value for each kmer (6-mer) in each read in the eventalign file and aggregated it by kmer. We then rounded to the nearest integer and generated normalized histograms representing the signal distribution in each kmer. We then used scipy.signal.find_peaks to identify kmers with a secondary peak in the positive control sample, which we considered to be an informative kmer to indicate modification. We also identified peaks in the negative control sample and found that no kmers had more than one peak in that sample (Supplementary File 1). Using these kmers, we then generated a sequence logo using kplogo (26) (http://kplogo.wi.mit.edu/).

### Scoring modification probability based on signal distributions

We generated precalculated modification probability scores given any informative kmer and any mean signal value (rounded to the nearest int) associated with that kmer. We did this by calculating the probability of any signal value belonging to the positive control distribution relative to the negative control distribution first by identifying whether the signal is closer to the secondary angelicin peak in the positive control data and then calculating the probability at that signal value of belonging to the positive control distribution relative to the negative control (pos probability / (pos probability + neg probability)). All kmers closer to the standard distribution were given a modification probability of 0.

### Aggregate analysis of transcription start sites (TSS) and transcription termination sites (TTS) using informative kmers

We took the eventalign data from yeast chromatin DNA and scored the modification probability of each informative kmer. We then aggregated those scores across reads for each genomic position and saved those in a .wig file. Next, we loaded all annotated TSS and TTS positions in the yeast genome (27). We went through the modification .wig file and aggregated all sites in relation to nearby TSS or TTS positions and plotted the average modification píobability at each position.

### Identification of missing kmers

To identify missing kmers, we first ran nanocompore eventalign_collapse (v1.0.4) (28) on our eventalign files for the negative and positive controls. We then went through the nanocompore collapse files and identified positions that were covered by aligned bases on either side within a read but do not have current signals assigned to them. We then aggregated these positions by kmers and compared the fraction of missing kmers to total kmers covered by aligned reads between the negative and positive controls.

### Training and validation of a neural network model

We developed a computational method NEMO (a NEural network model for mapping MOdifications in nanopore Long-read) designed for training and predicting angelicin modification sites. NEMO incorporates a PyTorch (v2.0.1+cu118) implementation of the Residual Network classifier tailored for analysis of one-dimensional signal data (29). We divided each positive and negative control dataset into training and validation subsets. Specifically, 80% of reads were allocated to the training dataset, while the remaining 20% were reserved for validation. Positive control data were labeled with prediction probabilities of 1.0 and negative control data were labeled with prediction probabilities of 0.0. The model was trained with an input size of 400, a batch size of 256 and 1000 batches per epoch. Input signals of length 400 are represented as a one-dimensional array [1, 2, 3, 4, 5, …, 400]. For every data point, a single signal shift was applied to capture the sequential nature of nanopore signals (e.g., [2, 3, 4, 5, 6, …, 401]). Gradient descent was computed using binary cross entropy loss after each step and model parameters were updated using the Adam optimizer (30). Following each epoch, model performance was validated with batch size of 256 and for 100 batches. After 500 epochs, the model with highest validation accuracy was saved as the optimal model for subsequent analyses.

### Neural network prediction in chromatin sequencing data

The model trained on control data was used for predicting angelicin modifications in chromatin sequencing data. Given a 75 base pair sequence, NEMO fetches corresponding signals, which theoretically matches 400 signals, and predicts angelicin modification in underlying bases. A sliding window of 75bp traverses across each individual read with a step size of 20 base pairs. This ensures neighboring predictions share information with 55 base pair overlap. Single-molecule modification scores were recorded for every 20 base pairs across the genome. Aggregated scores were calculated by averaging prediction scores across the reads. NEMO reports both single molecule scores and aggregated scores as final outputs.

### Single molecule clustering and visualisation

In NEMO, we implemented a matplotlib v3.6.2 based genome track visualizer to plot specific regions. Reads mapped to the *CLN2* gene promoter region chrXVI:66400–67480, *NUP170* gene promoter region chrII:74300–75800 and *ZDS2* gene promoter region chrXIII:51100–52600 are clustered and visualized using NEMO with following methods. Reads covering a minimum of 50% of the regions were used to construct a modification probability matrix. Missing values in the matrix were imputed with scikit-learn v1.1.2 simpleImputer function under ‘most_frequent’ strategy. The matrix was then input to the scikit-learn v1.1.2 K Means clustering algorithm, where reads are clustered based on their modification profiles. Clustering was performed with random centroid initializations and the cluster ids are collected after 300 iterations. Clustering numbers were decided based on previously reported numbers of clusters in literature (*CLN2*) or scikit-learn silhouette_score function (*NUP170* and *ZDS2*). Single molecules were colored based on their predicted angelicin modification scores. Modification scores were then binarized with threshold 0.55, and aggregated scores for each cluster were calculated by averaging binarized scores across the reads within each cluster.

## RESULTS

### Angelicin modification and sequencing of DNA

**Figure 1:**
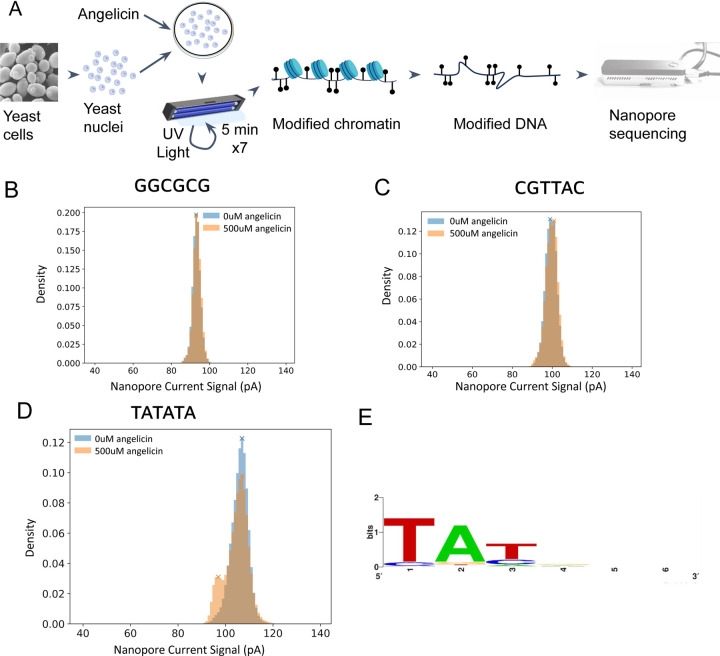
Add-seq: A method using angelicin modification to probe chromatin accessibility. (**A**) Schematic of the Add-seq method. Yeast nuclei were treated with 500uM angelicin, then exposed to multiple rounds of UV light to crosslink the angelicin with the DNA. The modified DNA was extracted and sequenced by nanopore sequencing. (Partly created with Biorender.com). (**B,C & D**) Histograms of the nanopore current signal data aggregated across all reads aligning to a given kmer from yeast DNA that had been either treated with UV light only or angeilicin + UV for (**B**) an unmodifiable kmer GGCGCG, (**C**) a modifiable kmer CGTTAC with only a single peak and (**D**) modifiable kmer with two peaks TATATA. (**E)** Sequence logo for the 58 kmers with two distinct peaks.

In order to obtain angelicin-modified DNA, we isolated yeast nuclei, preserving the chromatin structure, and incubated these with 500 uM angelicin (Figure 1A). We then exposed the nuclei with angelicin to UV-A (365nm) for seven rounds of 5 minutes each, allowing the nuclei to cool on ice between rounds. The time and rounds of UV-A exposure were previously optimized (3) for mapping nucleosomes by psoralen crosslinking to ensure high levels of covalent modification of DNA and minimize damage (Supplementary Figure 1). Following angelicin treatment of the nuclei, we extracted high-molecular-weight DNA to obtain single DNA molecules with a mean length of ~40 kb. We also extracted chromatin-free DNA from yeast nuclei and either treated it with only UV as our negative control or with UV + 100uM or 500uM angelicin as a positive control. We sequenced each sample DNA on an ONT minION (R9.4.1) flow cell, basecalled reads with Guppy v4.4.0, and aligned the current signal from the pore to the assigned genetic bases and genomic positions with eventalign.

While the modified DNA was successfully sequenced, we noticed that flow cells sequencing angelicin-modified DNA had significantly lower throughput and the pores became inactive significantly faster than flow cells sequencing unmodified DNA (Supplementary Figure 2A-B). Given that our preliminary structural analysis showed thiamine bases modified by angelicin could fit through a nanopore (data not shown), as well as the fact that we were able to sequence some angelicin-modified DNA, it was unlikely that angelicin intercalation was blocking the pores. Although previous work has shown that angelicin should form covalent bonds with a single thymine base on one DNA strand, without forming covalent bonds between the two strands of DNA (19, 20), molecules with structures similar to angelicin such as psoralen do cause crosslinking between DNA strands. To assess whether angelicin induces DNA crosslinking, we analyzed the mobility of angelicin-treated DNA samples in a denaturing alkaline agarose gel. This technique separates uncrosslinked single strands from cross-linked double strands based on their mobility (Supplementary Figure 2C). We found that angelicin treatment did cause a small fraction of the DNA to form interstrand crosslinks, which we hypothesized was causing the pore blockages. However, despite this reduced throughput, we were able to sequence and align 68,608 reads from the positive control sample modified with 500uM angelicin (Supplementary Table 1).

### Identification of angelicin modification from Nanopore current signal

Using the aligned current signal data from the positive and negative control samples, we compared the distribution of current signal values for 6 base-pair long kmers with and without the intercalation motif for angelicin (5’-TA). Kmers without 5’-TA had no shift in current signal values between the positive and negative control (Figure 1B), while a subset of kmers with TA had a secondary peak of different current signal values in the positive control (Figure 1D). However, most TA-containing kmers did not have any shift in the signal distribution between the negative and positive control (Figure 1C). Given previous work showing other modifications shifting current signals (14, 17) and that this shift was only observed in TA containing k-mers where the modification was possible, we concluded that this secondary distribution was due to angelicin-modified DNA.

To systematically distinguish angelicin-specific signal peaks of modified kmers from unmodified kmer signals, we identified distinct peaks in the signal distributions. Only 58/4096 (1.4%) percent of all kmers had this distinct signal shift. We then selected only kmers with 2 peaks in the positive control as informative. We then generated a sequence logo for these kmers, which showed a very strong preference for the known 5’-TA angelicin intercalation site as the first two bases of the kmer (Figure 1E). We were surprised not to observe signal shifts in kmers with TA at other positions.

We also observed that near a 5’-TA motif where we expect angelicin modification to occur, we instead observed positions with no signal mapped to the kmer (Supplementary Figure 3). These skipped kmers are positions where Guppy and Nanopolish eventalign could not assign kmers to the current signal in the read. The angelicin modification likely caused a shift in the electrical current that does not match the expected signal distribution of any known kmer, so the kmer is skipped by the software. 36% of all TA-containing kmers had >10% missing signal in the 500uM angelicin positive control sample. Modification probability cannot be assigned to these skipped kmers due to the lack of mappable signal to the sequence. Despite this sparseness of usable data, we modeled the probability of any signal value in an informative kmer belonging to the angelicin-specific peak in the signal distribution. In order to identify whether our data agrees with chromatin structure predictions from orthogonal methods, we used this model to predict the probability of angelicin modification at each informative kmer in the nanopore sequencing data from nuclei with intact chromatin treated with 500uM angelicin.

The region around a transcription start site (TSS) of a transcriptionally active gene typically shows a characteristic chromatin accessibility signal. Upstream of the TSS, the DNA is generally accessible allowing for transcription factors and RNA polymerases to bind. Downstream, within the gene body, nucleosomes are packed close to each other, so overall DNA accessibility is lower, and a regular pattern of positioned nucleosomes interspersed with accessible linker regions is expected. Furthermore, the first nucleosome is expected to be the most well-positioned, with subsequent downstream nucleosomes being less well positioned (31). Near the transcription termination site (TTS), there is also a known pattern of accessibility just downstream of the TTS (31). To see if angelicin-treated nuclei reflect this pattern, we averaged the modification scores for a 1000 bp window around every transcription start and end site in yeast (Supplementary Figure 4). From this metagene plot, we found that the scores roughly approximated the expected pattern around the TSS and TTS (27) with a peak of higher modification upstream and downstream of the TSS and TTS respectively. The dip in the NDR modification peak 5’ of the TSS possibly reflects the lack of informative kmers in this region.

### Identification of angelicin modification using a neural network model

**Figure 2.**
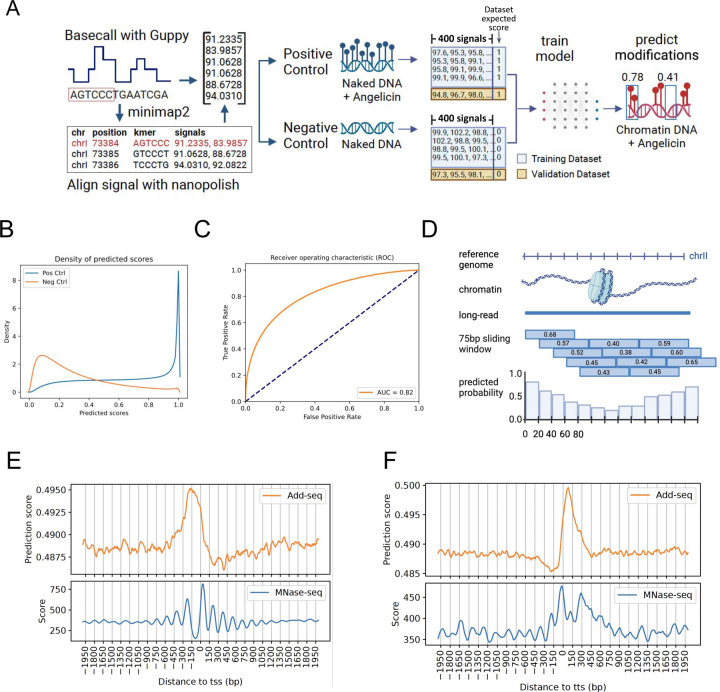
Angelicin modification scoring from a neural network model identifies expected patterns of chromatin accessibility around annotated gene loci: **(A)** A schematic of the neural network model trained on the untreated and angelicin treated DNA raw nanopore current signal data (Created with Biorender.com). **(B)** Density of predicted scores for negative and positive control data. **(C)** Receiver operating characteristic (ROC) curve for the validation set of positive and negative control data. **(D)** A schematic showing how modification probability is predicted for overlapping windows of 75bp on each read and then averaged to get scores for 20bp windows for each read (Created with Biorender.com). **(E & F)** Aggregate modification probability predicted by NEMO (top) and MNase-seq (bottom) for 4000 base pairs centered on every TSS (**E**) and TTS (**F**) in a subsample of the yeast genome.

Due to the low fraction of informative kmers and the missing kmers problem, the probability distribution model is only able to predict modification probability for 1.6% of genomic positions in the yeast genome. Given the difficulty in detecting modification at the single-nucleotide resolution, we hypothesized that chromatin accessibility could more easily be detected using machine learning to observe subtle changes in nanopore current signal across a larger window of bases. To be able to map single-molecule chromatin accessibility at nucleosome resolution, we developed NEMO (a NEural network model for mapping MOdifications in nanopore Long-read). To infer angelicin-modified regions, we trained a one dimensional residual neural network (ResNet1D) model directly from windows of consecutive nanopore signals(Figure 2A, Supplementary Figure 5) (32). ResNet1D has previously been used for monitoring electrocardiogram (ECG) signal data in intensive care units. Considering the analogous nature of electrical current measured by electrocardiograms and ONT flow cells, we think ResNet1D is ideal for learning signal changes caused by nucleic acid modifications. Since we were interested in being able to identify the presence or absence of nucleosomes, we picked a 400 signal measurement window, as that corresponds to approximately 75 bp or half of a nucleosome (Supplementary Figure 6). Our positive and negative control data were used to train and validate the classification ability of the neural network model. Our model was able to distinguish signal currents from positive and negative control data with an area under the receiver operating curve (AUC) of 0.82 in the validation dataset (Figure 2B, C). This represents a relatively high true positive rate and low false positive rate.

We then applied the model to predict accessible regions in nuclei-derived chromatin sequencing data. Individual reads are scanned using a 75 bp sliding window with a 20bp step size. Prediction scores are assigned to the first 20 bp within every 75 bp window by averaging overlapping windows (Figure 2D). After aggregating the neural network modification scores across all TSS and TTS, we observe the expected patterns of increased accessibility before the TSS and after the TTS. We observe the highest modification peak of angelicin immediately upstream of the TSS (Figure 2E) reflecting the canonical nucleosome depleted region (31) albeit with a broader angelicin modified peak than that observed with micrococcal nuclease. This is followed by a periodicity of angelicin modification roughly every ~150bp similar to the MNase-seq pattern of cleavage within the nucleosome linkers (27). The metagene plot of angelicin modification at the TTS (Figure 2F) shows high modification immediately following the TTS corresponding to the dual peaks observed in the MNase-seq plot.

**Figure 3.**
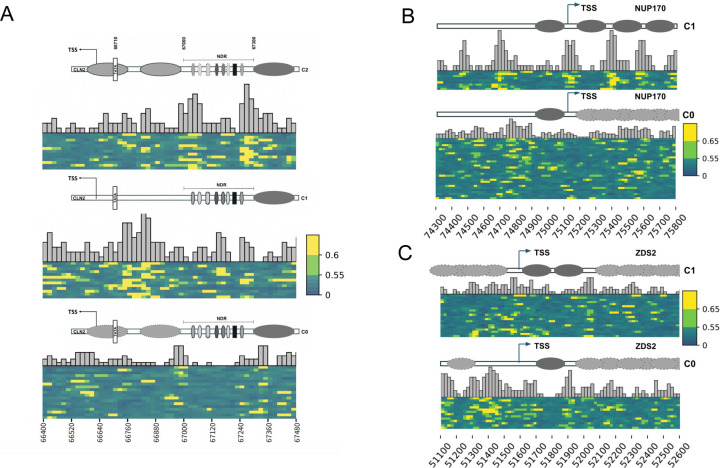
Single molecule analysis of chromatin structure using Add-seq: Single read angelicin modification clusters of individual gene loci. Each row is a single read covering the locus. Heatmap is probability of angelicin modification where 1 (yellow) is likely modified while 0 (green) is unlikely unmodified. The reads have been separated into clusters using k-means clustering on the modification scores. **(A)** The *CLN2* promoter (Chr XVI: 66,400–67,480). Dark gray ovals in the schematic represent the +1 nucleosome while the light gray ovals represent nucleosomes that are displaced by factor binding. The narrow vertical bars, ovals and hexagons represent transcription factor binding sites. **(B)** the *NUP170* TSS (ChrII: 74,300–75,800). Dark gray ovals in the schematic represent well-positioned nucleosomes and the light gray overlaid ovals represent overlapping nucleosome positions. **(C)** the *ZDS2* TSS (Chr XIII: 51,1000–52,600). Dark gray ovals in the schematic represent unique nucleosomes and the light gray overlaid ovals represent overlapping nucleosome positions.

Due to the higher density of modification calls in the neural network derived data, we also looked at individual loci. The *CLN2* cyclin regulates progression of the yeast cell cycle and transcription of the *CLN2* gene is also regulated in a cell cycle dependent manner (33). The *CLN2* promoter is a well-studied cell cycle regulated promoter that has a large nucleosome depleted region (NDR) upstream of the TATA box (34). Nucleosome depletion at the upstream NDR is achieved through the binding of cell cycle-specific and general transcription factors, and a chromatin remodeler complex (35). After k-means clustering of the predicted modification scores at this locus, we observe three clusters with distinct patterns of accessibility corresponding to a closed promoter with an open upstream NDR (C2), a partially closed promoter with a partially open upstream NDR presumably bound by some of the various factors (C0), and a fully open promoter with both nucleosomes displaced and a partially closed upstream NDR (C1) (Figure 3A). Alternative accessibility states at the *CLN2* promoter have been previously reported by (17), but we observe a more granular view of accessibility states that associates more closely with the known regulation of this promoter. The various *CLN2* promoter states that we can discern from our Add-seq approach demonstrate the usage of small molecules to study chromatin accessibility.

With our Add-seq single-reads, we are also able to observe the heterogeneity in nucleosome positions within a cell population that is mostly obscured in short-read data. The *NUP170* (YBL079W) TSS has been previously identified as having unique nucleosome positions using MNase-seq (36). In agreement with their finding, the *NUP170* C1 cluster in our data has a well-positioned nucleosome at the promoter followed by a uniquely positioned +1 nucleosome and fairly uniquely positioned +2 and +3 nucleosomes. Furthermore, we find a greater diversity of nucleosome positions within the gene between cells as well as a generally closed promoter in the second (C0) *NUP170* cluster (Figure 3B). The *ZDS2* (YML109W) gene has been identified as having overlapping nucleosome positions in the TSS using MNase-seq (36), which we were able to further resolve using Add-seq. As previously observed, there are two well positioned nucleosomes at the TSS for *ZDS2* in the C1 cluster and we find some overlap in nucleosome positions at the promoter of this gene. The C0 *ZDS2* cluster on the other hand, reveals a mostly open promoter with a well-positioned +1 nucleosome (Figure 3C). These observations highlight the diverse positions nucleosomes occupy within a cell population and support the utility of Add-seq in analyzing chromatin structure.

## DISCUSSION

We have established that angelicin can covalently bind to thymine bases in single strands of DNA and that those strands can be sequenced on nanopores. We have also shown that angelicin-modified kmers have a distinct current signal compared to unmodified kmers. We have developed a neural network-based method for estimating the probability of angelicin modification on segment of DNA. These methods allowed us to detect both the chromatin accessibility on a genome-wide level as well as at the single-molecule level at specific loci. The biggest challenge we have faced is the sparseness of data likely due to a combination of incomplete angelicin modification of accessible modifiable sites, blockage of the pores presumably due to DNA cross-linking, and the reduced ability of the base calling software to consistently assign DNA sequence to modified kmers. Some of the incomplete angelicin modification may be because at any AT/TA context, angelicin can only covalently bond with a thymine in one strand of the DNA. As a result, we fail to sequence the strand containing the modification half the time with standard nanopore sequencing. This means that even in our positive control, we do not have any guarantee that all modifiable sites will be modified. This is a non-trivial problem especially for the neural network-based model, as machine learning models depend highly on good-quality training data. Other groups have used synthetic DNA with modified bases at known sites to train similar models. However, we were unable to find any available protocols or companies able to synthesize DNA with angelicin modifications. Future work may utilize the newly developed nanopore duplex sequencing to sequence both strands of DNA (37, 38), increasing the probability of sequencing the modified kmer at each modifiable position. However, at the moment, this method is not high throughput enough to generate sufficient training data (37, 38).

Although previous work has shown that the chemistry of angelicin should not allow for the formation of crosslinks (19, 20), unlike the angelicin analog psoralen that only forms interstrand crosslinks (3), we observed a small fraction of angelicin treated DNA contained interstrand crosslinks. This result combined with the more rapid decay of flow cell pores on samples with angelicin treatment leads us to hypothesize that the interstrand crosslinks in the DNA cannot pass through the pores, thus clogging them and reducing the throughput of the flow cell. One way to alleviate this issue would be to incubate DNA at elevated temperature and basic pH to break interstrand crosslinks. Base treatment has been successfully used before to break DNA crosslinks formed by psoralen (39) but the adapter protein required to ratchet the DNA through the nanopore during sequencing will not withstand such harsh treatment thus precluding this option. Other options include modeling angelicin itself as well as altered structures of angelicin or other furocoumarin derivatives to determine how they traverse the nanopore and utilizing this information to synthesize and test alternative small molecules that can be used as probes for visualizing the chromatin landscape (40, 41).

Despite these challenges, our current protocol still allows us to detect both genome-wide and single locus chromatin accessibility. There are also other benefits to using this small molecule as opposed to methylation enzymes or other approaches. Compared to enzyme-based approaches, angelicin modification is significantly cheaper per unit of DNA- $0.09 per ug of DNA for angelicin compared to $4.30 per ug of DNA for the commercial EcoGII methyltransferase. Angelicin is also an exogenous modification that does not naturally exist in cells. Other approaches use GpC methyltransferases to label genomes that also have endogenous CpG modification, which results in the exclusion of methylation data in a GCG context due to ambiguity between native methylation and exogenous modification (14). Angelicin is also a membrane permeable molecule, which can facilitate chromatin accessibility probing without isolating nuclei (19), which has been previously shown to affect chromatin structure accessibility (42). Removing the step of nuclei isolation can make accessibility probing more amenable to small tissue samples or other single-cell analysis. Furthermore, DNA polymerase is unable to polymerize through an angelicin modified template (data not shown); therefore, this technique of detecting accessibility will unlikely work with other long-read sequencing methods like Pacific Biosciences (PacBio) (15). While there are still optimizations that could be made to the angelicin modification protocol, we show that using nanopore sequencing on angelicin-modified chromatin is a novel method for probing chromatin.

## Supplementary Material

Supplement 1

Supplement 2

## Data Availability

Raw nanopore signal data are deposited at https://zenodo.org/records/10798988. Basecalled nanopore sequencing data and alignment files are available under BioProject: PRJNA1084879. Data and Codes for regenerating figures are at: https://github.com/baigal628/addseq_manuscript. Our computational model NEMO is available at https://github.com/baigal628/NEMO. Processed data is available in Supplementary File 1.
