# Supplementary material for: Probing chromatin accessibility with small molecule DNA intercalation and nanopore sequencing
Source: bioRxiv. 2024 Mar 22:2024.03.20.585815. Preprint. [Version 1] doi: 10.1101/2024.03.20.585815 (PMC10983977; doi:10.1101/2024.03.20.585815)
Supplement: Supplementary file 1 [file NIHPP2024.03.20.585815v1-supplement-2.pdf]

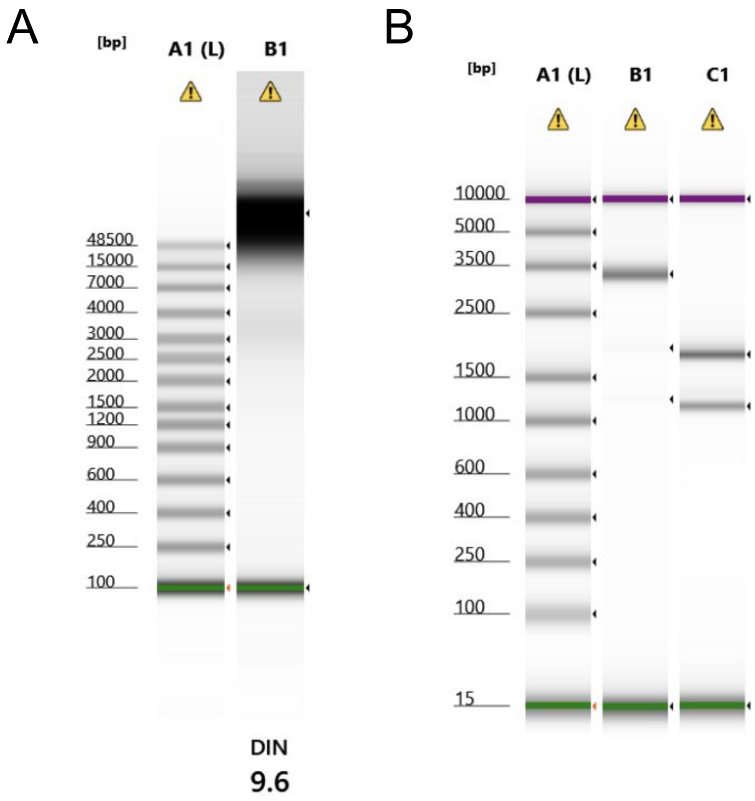

Supplementary Figure 1. Quality of Angelicin modification of DNA:
(A) TapeStation results for 500uM angelicin treated DNA after treatment with short read eliminator (SRE). SRE was not used in sequencing because it did not improve the result.
B Result of BciV1 digest on 500uM angelicin sample (lane B1) and 0uM angelicin sample (lane C1). BciV1 cuts at TA sites and fails to cut when angelicin modification is present at these sites.

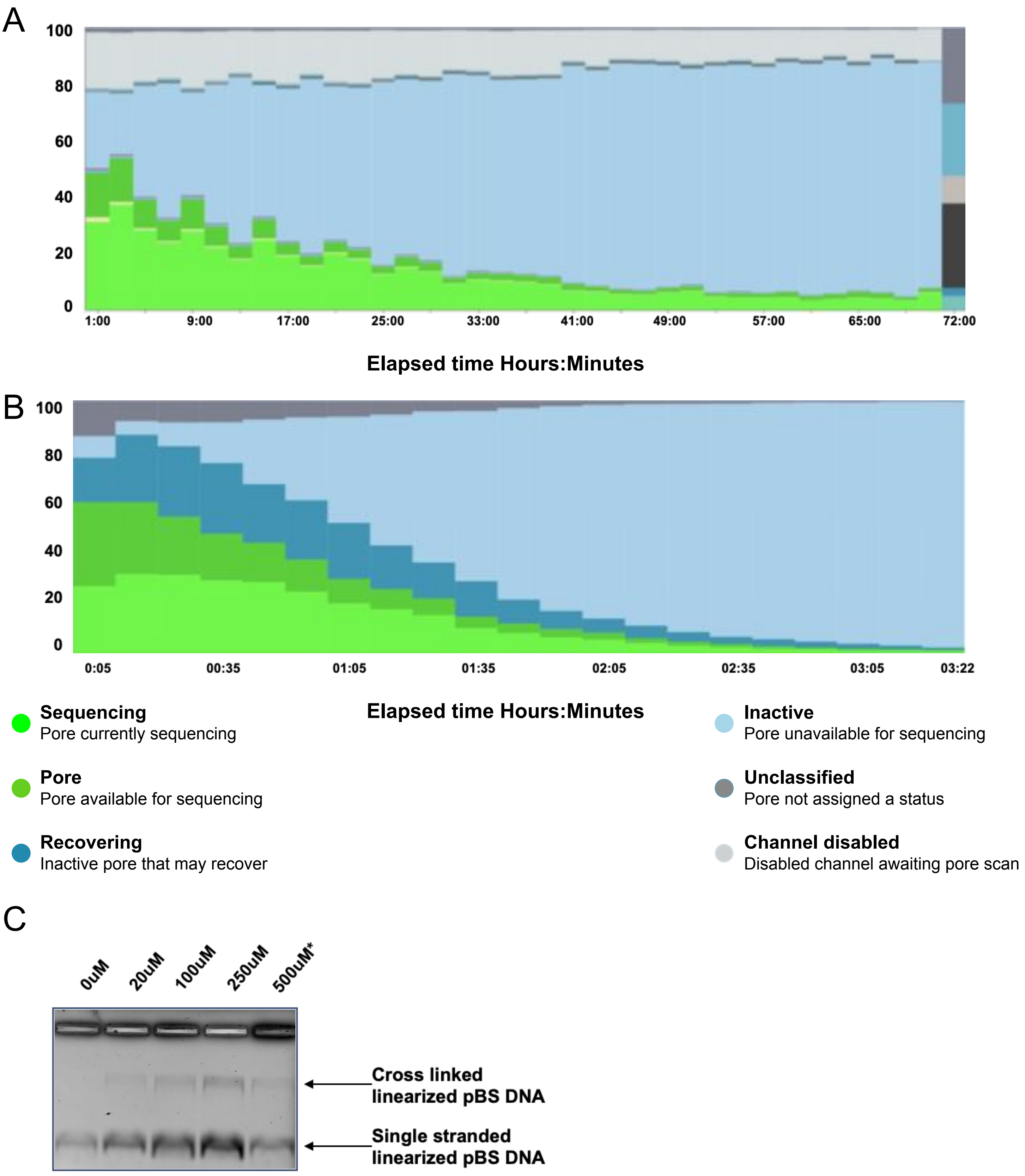

Supplementary Figure 2. DNA crosslinking due to angelicin modification leads to reduced throughput of flow cells: (A+B) The histograms show percent activity of nanopores during a sequencing run with **A** Unmodified DNA, X axis has a maximum value of 72 hours and **B** DNA modified with 500uM angelicin, X axis has a maximum value of ~3 hours. (**C)** Denaturing alkaline agarose gel electrophoresis of linearized plasmid BlueScript (pBS) modified with varying concentrations of angelicin (0uM to 500uM). A majority of the DNA migrated as single stranded DNA (lower band) however, a small amount of double stranded DNA (upper band) was observed in lanes with angelicin modified DNA. A portion of the 500uM* angelicin treated DNA was lost during processing.

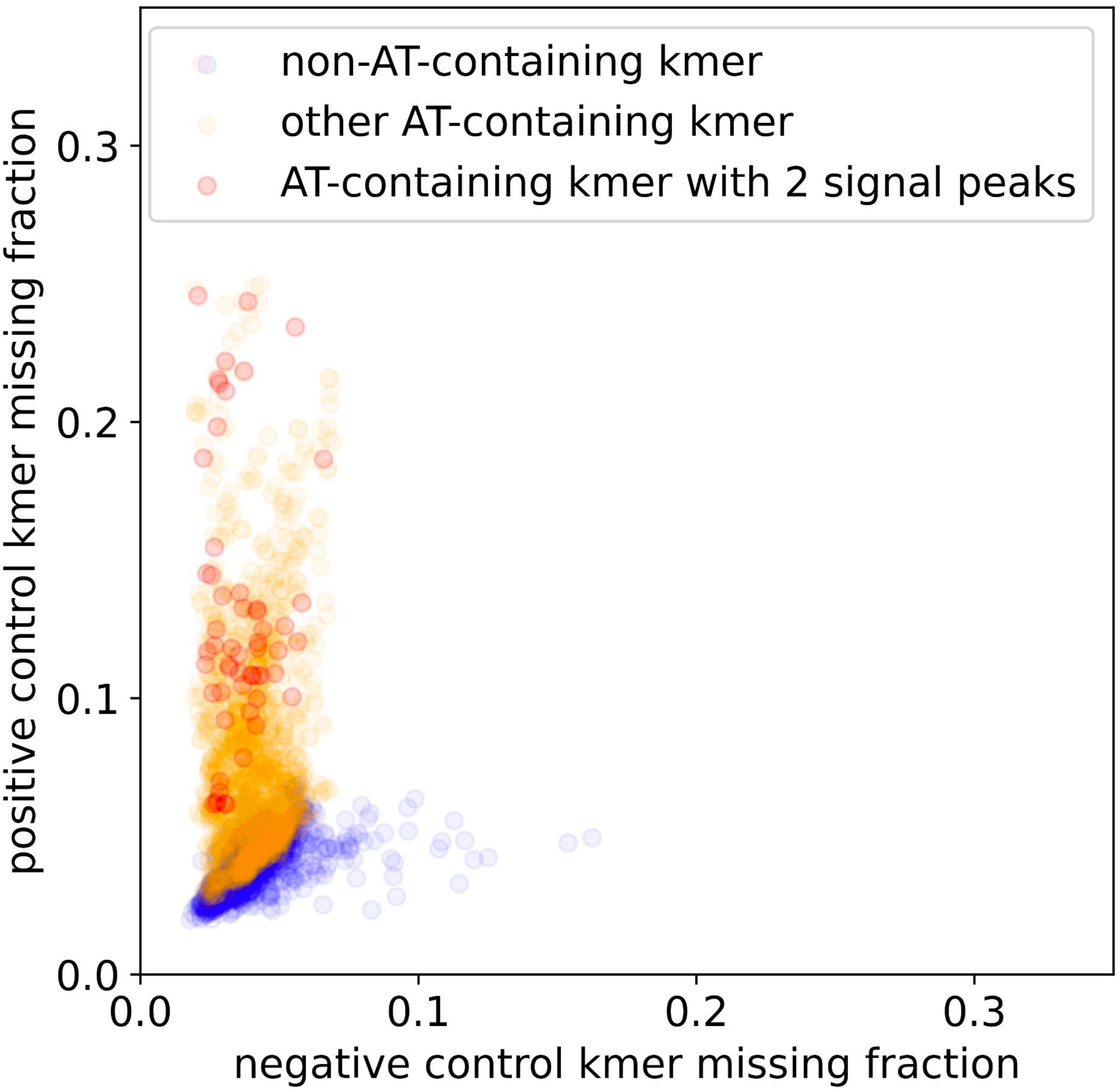

Supplementary Figure 3. Missing kmer analysis: Points are kmers covered by reads but with no signal aligned to them in the negative vs positive (500uM angelicin) control samples.

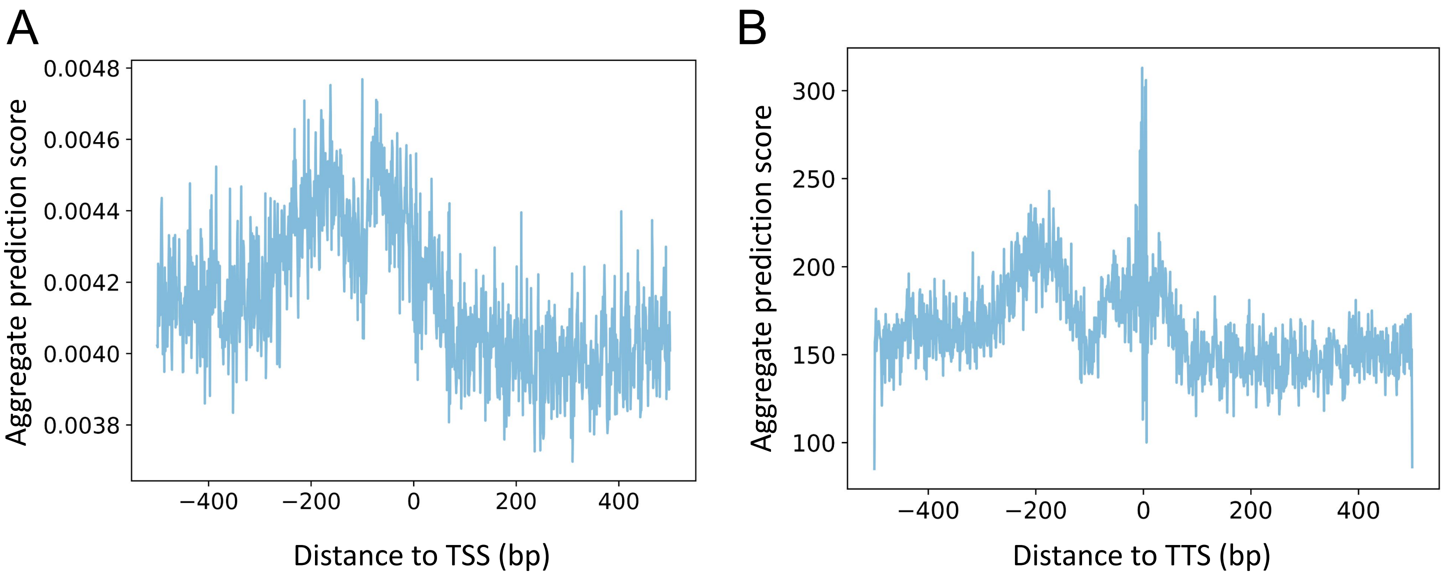

Supplementary Figure 4. Aggregate accessibility prediction using statistical model on only informative kmers: A TSS and **B** TTS aggregate scoring for statistical prediction of kmer modification for yeast DNA with chromatin and angelicin modification. Statistical prediction was performed only for the 58 informative kmers.

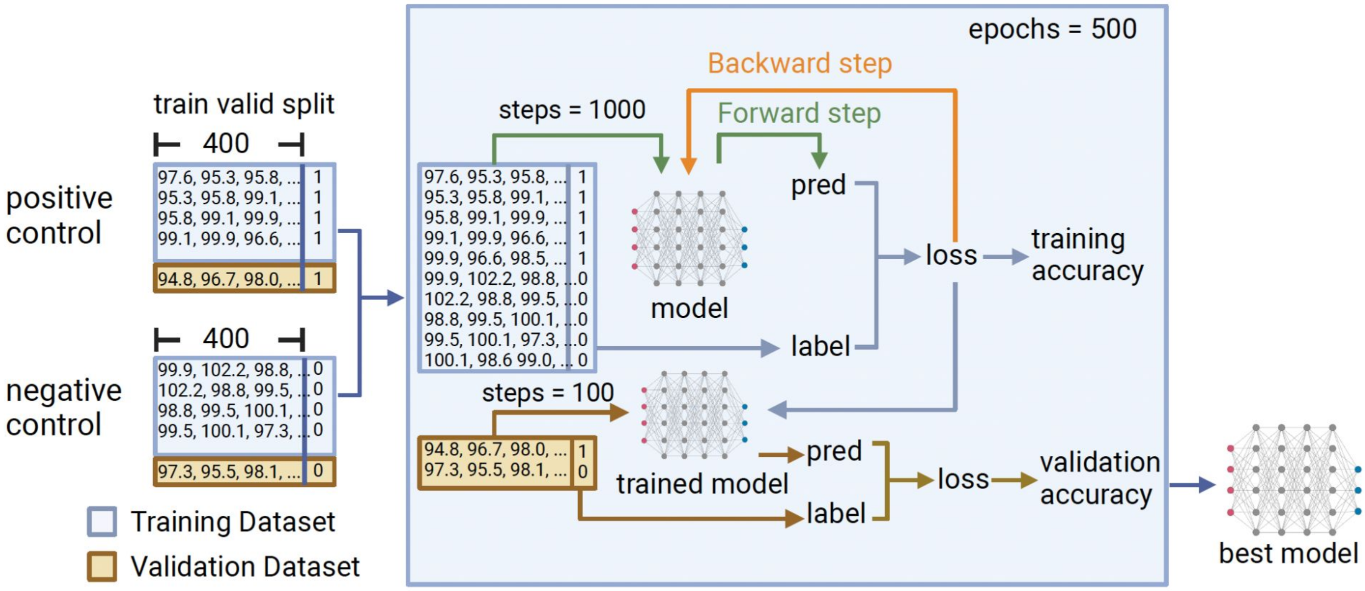

Supplementary Figure 5. Expanded schematic of neural network process. Created with Biorender.com

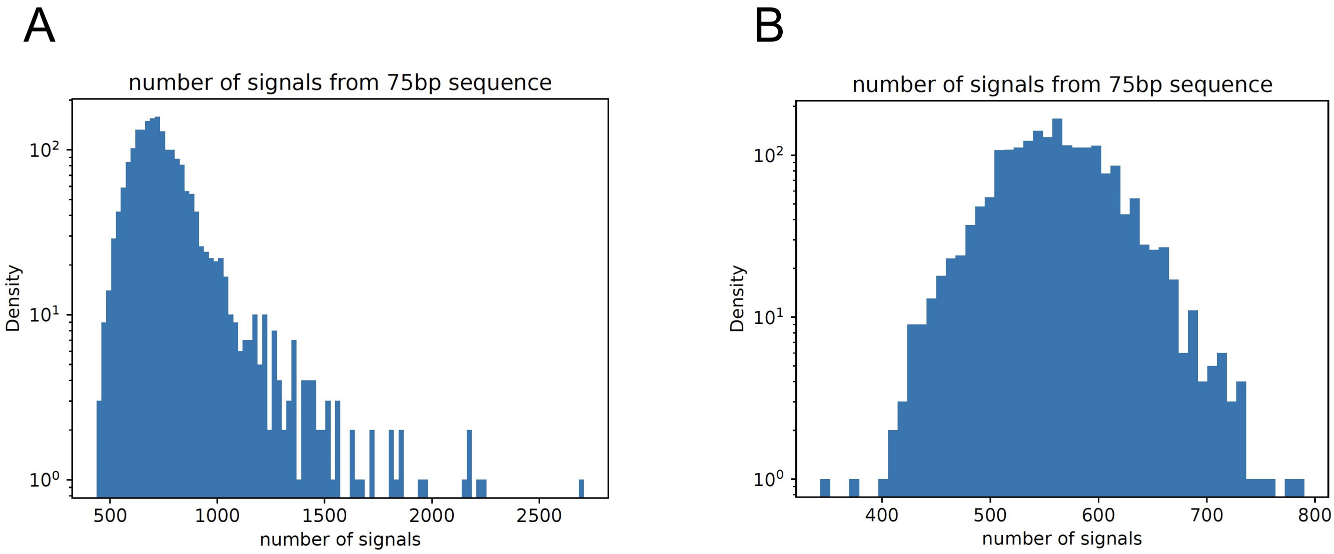

Supplementary Figure 6. Histograms of signals associated with a 75bp window: (A) Number of signal scores per 75bp window **(B)** Number of signal scores per 75bp window after eliminating 6-mers with > 10 scores assigned to them (indicates DNA pausing, causes outliers)

SAMPLE	Number of Raw Reads	Total raw bases	% Aligned reads	Aligned bases excluding mismatches	Fraction of aligned + matched bases	Average coverag e	Average base quality	N50 Length (kb)
**Negative Ctrl**	*76,833*	*351,075,059*	95.5%	320,253,441	91.2%	27.6X	22.4	*16.2*
**Positive Ctrl 100uM**	*51,309*	199,635,317	92.4%	170,192,907	85.2%	15.1X	20.0	*10.6*
**Positive Ctrl 500uM**	121,848	377,864,115	89.0%	292,057,136	77.3%	26.6X	18.3	*6.76*
Chromatin **500 uM**	*108,470*	815,894,534	92.2%	713,990,306	87.5%	62.8X	21.1	*13.4*

Supplementary Table 1. Sequencing and alignment statistics for Nanopore sequencing of all samples
